# Systemic therapy informed self-help and paraprofessional training in a naturalistic setting: study protocol of a digital randomised trial

**DOI:** 10.1186/s13690-025-01597-1

**Published:** 2025-05-06

**Authors:** Pieter Erasmus, Moritz Borrmann, Ayesha Tariq Ali, Stefanie Herta, Jule Becker, Glen Moriarty, Gunther Meinlschmidt

**Affiliations:** 1 Division of Clinical Psychology and Cognitive Behavioural Therapy, International Psychoanalytic University (IPU) Berlin, Berlin, Germany; 2 Day Clinic for Cognitive Neurology, University Hospital Leipzig, Leipzig, Germany; 3 7 Cups, Palo Alto, CA USA; 4 Government College University Faisalabad (GCUF), Faisalabad, Pakistan; 5 Department of Digital and Blended Psychosomatics and Psychotherapy, Psychosomatic Medicine, University Hospital and University of Basel, Basel, Switzerland; 6 Department of Psychosomatic Medicine, University Hospital and University of Basel, Basel, Switzerland; 7 Department of Clinical Psychology and Psychotherapy – Methods and Approaches, Trier University, Trier, Switzerland

**Keywords:** Systemic psychotherapy, Paraprofessional training, Self-help, Digital mental health, Intervention, Psychosocial distress

## Abstract

**Background:**

Multiple studies have shown the efficacy of Systemic Psychotherapy (ST) approaches as a promising path to address various mental health disorders and alleviate psychological distress. One promising path that requires further investigation is online self-help interventions and paraprofessional training. Our study aims to evaluate the implementation of delivering ST-informed online self-help interventions (OSI) and paraprofessional training in a naturalistic setting via the 7 Cups platform.

**Methods:**

Our exploratory, randomised, controlled, pre-post-follow-up study is recruiting two samples of participants: (i) for the ST-informed online self-help/growth path intervention amongst members on the 7 Cups platform, (ii) for the ST-informed online paraprofessional/listener training intervention amongst members who have registered as ‘listener’ status. The sample size target is *N* = 200 participants, randomly assigning and comparing 50 OSI member participants to 50 waitlist member participants and 50 paraprofessional/listener training listeners to 50 waitlist listener participants. Our sample size justification, based on previous feasibility studies and guidelines, suggests that a sample size of 50 per group, accounting for a 25% attrition rate, is sufficient to assess feasibility in online family, e-health, and mental health trials, while maintaining narrow margins of error for confidence intervals and primary outcome parameters. The primary outcome is non-specific psychological distress (Kessler Psychological Distress Scale; K6); secondary outcomes include perceived stress, anxiety and depressive symptoms, and momentary mood. To estimate intervention effects, we intend to conduct generalised linear mixed models of primary, and secondary parameters, adjusted for potential covariates (e.g., gender, age categories, socioeconomic status) as fixed effects.

**Discussion:**

Despite the great need for mental health interventions, there is limited research available showing the efficacy of digitally delivered ST-informed interventions. This exploratory, randomized, controlled trial will yield robust insights into the feasibility, challenges, and user experience of novel, digital interventions on a widely accessible online platform (7 Cups).

**Trial registration:**

ClinicalTrials.gov (NCT06017414, updated 21.11.2023). Registered 29 August 2023, https://clinicaltrials.gov/study/NCT06017414.

**Supplementary Information:**

The online version contains supplementary material available at 10.1186/s13690-025-01597-1.

**Table:** 

**Text box 1. Contributions to the literature**	
• This study protocol addresses a gap in the public health literature by evaluating a digital, systemic therapy-based self-help and paraprofessional program.	
• It explores how training non-professionals alongside guided self-help can expand and strengthen mental health support globally, reaching people who lack access to traditional services.	
• The findings may help guide future research and practice on integrating systemic therapy principles into digital mental health strategies to reduce psychological distress on a large scale.	

## Background

The efficacy of family and parenting interventions is well-established for treating various mental health disorders [1–3]. Multiple studies have shown the efficacy of these interventions delivered digitally [4, 5]. However, definitions of family and parenting therapies can often be broad and defined more by the setting than the content of the intervention. Systemic Psychotherapy (ST) contains multiple elements of family and parenting interventions and provides a more unified definition, characterised by the content of the intervention rather than the setting. In recent years, ST has received an increasing amount of attention and acknowledgement, such as the 2017 report by the *Institut für Qualität und Wirtschaftlichkeit im Gesundheitswesen (IQWiG)*, a comprehensive systematic review outlining the efficacy and effectiveness of ST in treating the most common mental health disorders [6], the official declaration and integration of ST into the German public healthcare system in 2018 by the *Gemeinsame Bundesausschuss (G-BA)*, as well as various other comprehensive systematic reviews and meta-analyse [7, 8]. While individual definitions of ST differ, there are certain characteristic core elements and principles that allow for a largely agreed-upon and usable definition based on the following principles and techniques: an emphasis on relational and social systems, neutrality or multipartiality, focusing on individuals’ and systems’ resources, recognizing interpersonal patterns that contribute to psychological distress, and techniques such as joining, contracting, linework, witnessing and sculpture work [9–12]. Various systematic reviews (e.g. Carr [2], IQWiG, [6]; Riedinger et al., [7] highlight these components as characteristic of ST and fundamental in distinguishing it from individually oriented treatments. In this study, we integrate these principles and techniques in both the self-help and paraprofessional training programs, thus referring to them as ST-informed [6, 12]. For research and practice, ST can be characterised as “intervention into complex human systems (both, psychological and interpersonal) with the aim of reducing or eliminating suffering” [13] (translated by MB). ST can further be defined as a conceptual framework for mental health interventions that incorporate interpersonal relations, social interactions, perspectives, constructions of situations and problems, and appreciating and utilising attempted solutions as an integral part of the intervention [7, 9, 14]. Although systemic therapy is often delivered to couples, families, or other relational systems, it is also the case that individual-focused ST adaptations can be used to leverage systemic principles in an individual context (e.g., encouraging an individual to explore relational patterns and multi-perspective taking) [2, 6, 15]. Our digital modules similarly focus on these relational perspectives and interactions, even though each participant completes the program individually. Although research has supported the efficacy of ST interventions, further research is required. It is of crucial importance to critically evaluate ST interventions to avoid potential *culs-de-sac*, as well as to identify the most promising paths for efficacious interventions and ‘’conditions under which systemic therapy works best’’ [7], (p. 881).

Similar to digitally delivered interventions of family therapy interventions [16, 17], digitally delivered ST in the form of online self-help interventions (OSIs) may be one such promising path. This approach is particularly promising as digital delivery continues to advance and may help to overcome some of the common impediments to the worldwide delivery of psychotherapy such as limited access [18, 19], cost, and stigma [20]. While ST has been validated primarily in clinical populations, data suggest its approach may also benefit non-clinical users who experience stress or mild psychological distress [2, 5, 21–23]. Because 7 Cups members and listeners do not necessarily have formal diagnoses, this study seeks to determine if an ST-informed approach can be feasibly applied and beneficial to a broader population, including subclinical or undiagnosed distress. OSIs can be broadly divided into guided and unguided modes of self-help, with the former showing better efficacy than the latter [24]. This distinction notwithstanding, unguided OSIs are effective in the treatment of anxiety [25], depression [26], the prevention of suicides [27], and numerous other mental health disorders and conditions [28]. From a public health perspective, OSIs are particularly interesting as they promise to address several obstacles to conventional mental health care provision and harness patients’ capacities for self-help [29]. Additionally, their scalability might facilitate the provision of services to target groups that could otherwise not be reached [30]. While there still appears to be some level of discrepancy in terms of perceived acceptability in patients and practitioners (the latter rating acceptability lower than the former) [28], OSIs are particularly effective within so-called “blended care” approaches, integrating OSIs with face-to-face treatment [31]. Another way in which OSIs might prove a useful addition to existing modes of treatment is within a so-called “stepped care” approach where OSIs function as a first “step up” following prevention and diagnostics and preceding other forms of treatment such as face-to-face psychotherapy [32]. Platforms like “7 Cups” (https://www.7cups.com) offer self-help, text-based paraprofessional support, and signposting to professional services, which aligns with some recommendations and prior research in digital mental health research on the 7 Cups platform [1]. Further, systematic reviews indicate that platforms combining self-guided digital content with human support can be especially promising (see Sin et al. [33] for a general review of digital interventions). Although systemic therapy is commonly delivered to couples, families, or other relational systems, it is also the case that individual-focused ST adaptations can be used to leverage systemic principles in an individual context (e.g., encouraging an individual to explore relational patterns and multi-perspective taking). Our digital modules similarly focus on these relational perspectives and interactions, even though each participant completes the program individually. Our study focuses on the feasibility and exploratory efficacy of such an approach, rather than making conclusive claims about its effectiveness.

There is, to our knowledge, only one published study on an OSI explicitly incorporating methods and techniques associated with ST [1]. There is thus a substantial gap in the current literature. In addition, platforms such as 7 Cups, whose peer support programme has received some attention, warrant further scrutiny. For example, attrition related to the OSIs offered by platforms such as 7 Cups (https://www.7Cups.com) is a newly emerging field of research [34] to which our study could make a timely and meaningful contribution. The integration of paraprofessionals (called listeners on the 7 Cups platform), as service providers promises to provide a potential solution to the aforementioned issue of attrition and lack of engagement while retaining the scalability of OSIs. Torous and colleagues [35] identified the addition of human support and human feedback as two potential key factors reducing dropout and increasing engagement, which however warrants further study. Additionally, a recent meta-analysis indicated that paraprofessional guidance for OSIs improved effectiveness outcomes compared to unguided OSIs and was not significantly different from professional guidance [36]. While the integration of human support and human feedback might negatively impact the scalability of OSIs [35, 37], this impact might be less substantial for paraprofessional service providers such as listeners on 7 Cups, as opposed to trained mental health professionals [36]. Training paraprofessional service providers is studied in a variety of contexts and settings such as universities [37], underserved populations [38], or low- and middle-income countries [39]. While it is acknowledged as an important part of improving access to mental health care globally [40], there are to our knowledge no studies examining training paraprofessionals in ST techniques on a widely accessible online platform such as 7 Cups. We refer to “paraprofessionals” (or “listeners”) as volunteer lay people who have completed a basic training in active listening offered by 7 Cups. Although they do not function under ongoing, real-time supervision by licensed mental health professionals, 7 Cups maintains internal oversight, guidelines and training, channels to escalate services offered from lay people to professionals, and escalation procedures for safeguarding, ensuring that critical situations are referred to appropriate services. Listeners and professionals can be accessed through the same platform and can be considered to work alongside each other.

## Objectives

The main goal of this project is to address several gaps in the current literature in an exploratory way. This project seeks to have a meaningful impact on three fields of research:

1. The digital implementation of principles and approaches of ST into unguided self-help growth paths.
2. The training of paraprofessional mental health service providers with listener training.
3. The utilisation of paraprofessional mental health service providers in the delivery of online guided self-help interventions.

### Research questions

This study aims to address the following research questions:

1. Are OSIs based on ST approaches and principles feasible?
2. Are OSIs based on ST approaches and principles efficacious (as compared to a waitlist), indicated by mental-health-related outcomes of participants?
3. Is training of paraprofessionals in guiding the use of ST approaches feasible, and does such training improve their own mental health-related outcomes (as compared to a waitlist)?

Additionally, we seek to generate exploratory data on the following question using qualitative exploration:

- How does the guidance by paraprofessionals trained in ST-informed approaches and principles affect the experience and outcomes of participants doing the ST-informed OSI?

## Methods and design

### Overview of research project and study design

The main components of our study are:

i) An exploratory, randomised, controlled trial following the definition of the National Institute for Health Research UK (NIHR UK) in a natural setting, pre-post-follow-up design with two parallel comparisons:
  - the 2 arm ST-informed OSI (growth path) intervention compared with a waitlist comparator condition among 7 Cups members, and,
  - the 2-arm ST-informed paraprofessional provider/listener training compared with a waitlist comparator condition among 7 Cups listeners.

**Fig. 1:**
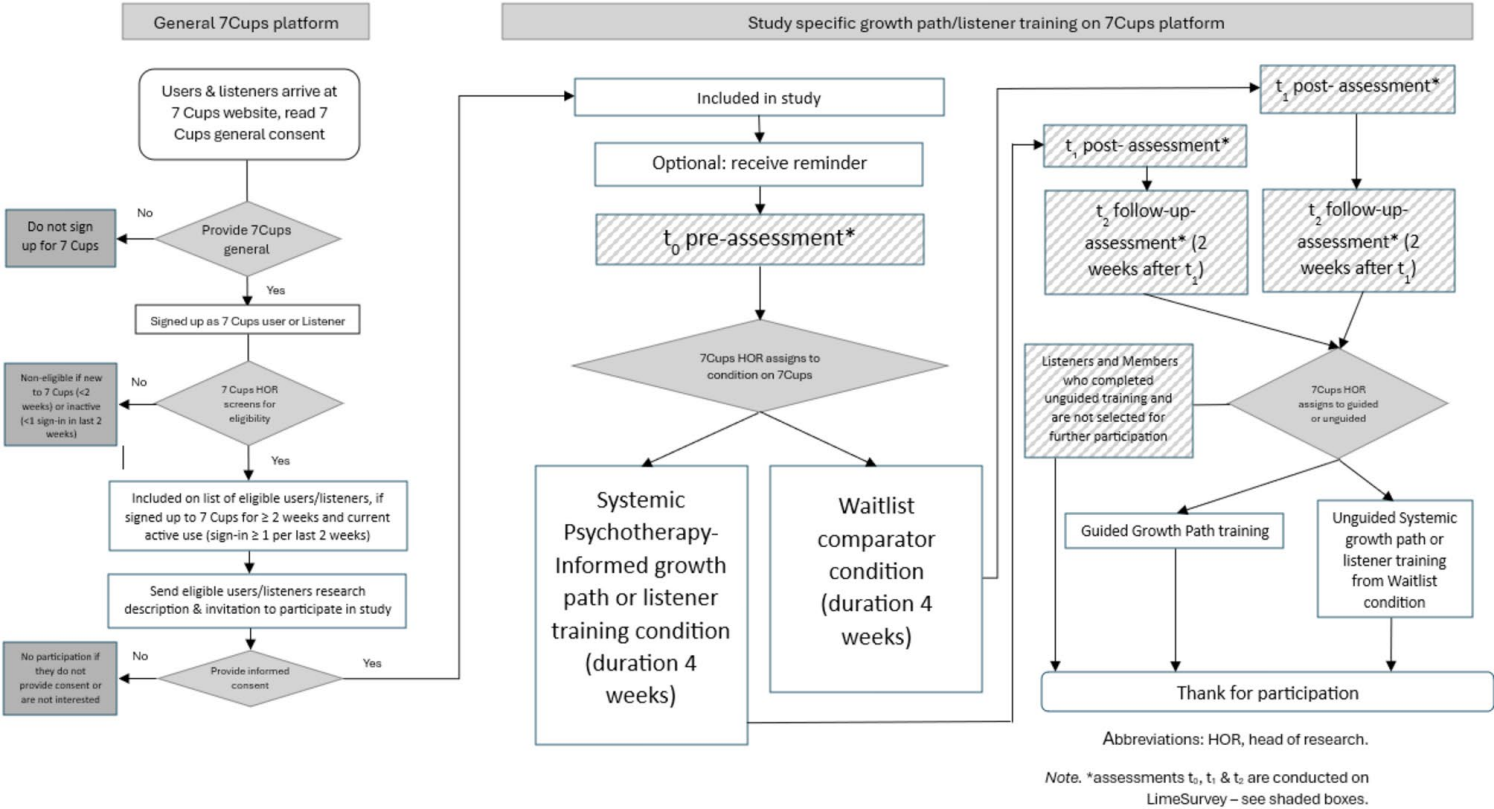
Outline of study design and flow of study participants

Each comparison uses a 1:1 allocation ratio. Figure 1 displays participant flow in each sub-study, including randomization to intervention vs. waitlist and subsequent follow-up assessments. Although this is not a fully powered randomized controlled trial (RCT), randomization will be used to test the procedure and to gather exploratory efficacy data.

The design shall facilitate the examination of the exploratory primary and secondary research questions and was deemed practical for the naturalistic setting.

### Participants, interventions, and outcomes

#### Study setting

The study is conducted on the 7 Cups platform, an online community connecting people with emotional support features via a website (www.7Cups.com) and application. This platform is accessible to individuals worldwide, and data are collected from multiple countries eligible to use the 7 Cups platform.

#### Participant selection

Our study participants involve users on 7 Cups, with two types of users: members and listeners. These individuals may or may not have a diagnosed mental disorder, but they are not currently experiencing a crisis. Users seeking support, also called members, often face emotional and psychosocial distress. It’s important to note that 7 Cups is not intended to be used by anyone currently experiencing crises. Upon signup, users confirm they are not in crisis and agree not to use the platform for such situations. For crises, 7 Cups provides resources and alternative channels for help. Support on 7 Cups comes from trained paraprofessionals known as listeners. Paraprofessionals, referred to as listeners, provide support to the members or users of 7 Cups and are expected to be on the lower threshold of psychological distress compared to members.

#### Inclusion criteria

To be eligible for participation in our study requires being signed up on the 7 Cups platform as either a member or a listener. Members are defined as ​​individuals seeking support and looking to discuss their concerns. Listeners are paraprofessionals trained on the platform in active listening techniques to provide emotional support to members. For both members and listeners, the following eligibility criteria apply: (1) they have used the 7 Cups platform by logging in at least once in the last 2 weeks, (2) they are aged 18 years or above, (3) they agree to participate in our study and provide consent to data usage, and (4) they have sufficient English language proficiency to interact on the 7 Cups platform, participate in the intervention, and complete the assessments.

#### Exclusion criteria

Exclusion results from the refusal to consent to data use.

### Interventions

#### Structure of the ST-informed self-help (growth path) intervention

The growth path intervention is designed as a four-week, module-based program delivered on the 7 Cups platform. Participants can complete it at their own pace within approximately 4 weeks. The program comprises five modules with each taking approximately one week to complete except for the first module which is a brief onboarding module. Each week participants are introduced to ST principles and techniques [10, 11], namely joining, contracting, linework, witnessing, and sculpture work. Each module includes psychoeducational text, reflection prompts, and self-directed exercises. Joining focuses on the start of the process with particular emphasis on selecting and writing down a personal situation, with guidance on goal setting, non-judgmental attitudes, and motivation to participate. Contracting provides an informal contracting process that aims to foster commitment to complete the program within the 4-week timeline. Linework encourages the exploration of personal relationships through drawing exercises that encourage perspective taking in the context of a relevant salient situation and relationship, and as illustrated with lines and figures. Witnessing guides participants in imagining trusted relationships to observe their situation, interactions, and emotional responses from multiple perspectives. Sculpture work prompts participants to visualize and enact their situation with the use of physical objects, to explore relationship structures, and dynamics through guided acting exercises. The techniques are repeated with increasingly elaborate instructions. For example, with ‘’linework’’, start by drawing a circle that represents yourself, then draw a circle that represents your situation, then draw a line to represent the connection between the two circles. The intervention is standardised and monitored via proxy of the pre and post assessment completion dates, with reminders sent to participants that have not reached the end of the program as indicated by completing (or declining) the assessment.

#### Structure of the ST-Informed paraprofessional (Listener) training

The paraprofessional training intervention is closely aligned with the OSI content. It also consists of five modules and is designed as a four-week program. Each module introduces the same ST-informed principles and techniques, followed by additional guidance for paraprofessionals. These listeners are prompted to complete the reflective exercises themselves and then to consider how they might support members encountering similar activities. For example, in the ‘’witnessing’’ module, listeners practice perspective-taking in their own relationships and situations, reflect on what the experience would be like and where they can support a member, and subsequently learn to encourage members to reflect on different perspectives of a shared situation using the witnessing exercise. The intervention is standardised and monitored via proxy of the pre and post assessment completion dates, with reminders sent to participants that have not reached the end of the program as indicated by completing (or declining) the assessment.

#### Waitlist condition

Participants in the waitlist condition do not receive access to the ST-informed modules. They continue to use the general features of the 7 Cups platform (e.g., chat rooms, peer-support, standard listener responses, and forums) without receiving the specific ST-informed self-help or training material. To reduce contamination, active-condition participants are instructed not to share the material in any of the public or group spaces. These spaces are monitored and in any cases that links, exercises, or content is shared, the 7 Cups head of research (HOR) and their team removes them. Waitlist participants are granted full access to the ST-informed modules only after completion of the final (t2) assessment or upon withdrawal from the study (if they wish to access it after withdrawal).

Participants are sent reminders to encourage them to continue and complete the intervention and assessments every two weeks and the post assessment after 4 weeks. Adherence rates are measured and shall be included in the analysis as an indicator of the feasibility of the intervention. Participants in our study are free to use any concomitant support or interventions on the 7 Cups platform or elsewhere. Waitlist participants continue their usual use of 7 Cups, without any access to ST-informed intervention content during the waitlist phase. Waitlist participants are requested to report the use of any other growth paths on 7 Cups in the post assessment. Both conditions use 7 Cups as normal, but the active conditions have access to the ST-informed learning content that is not available elsewhere on the 7 Cups website.

#### Ancillary and post-trial support

We do not provide systematic ancillary and post-trial support, yet in case of such need, patients can direct themselves to multiple resources made available on the 7 Cups platform.

### Outcomes

Our outcomes are divided into primary and secondary outcomes. We provide a full list of assessment instruments in Table 1 which also includes the list of primary and secondary endpoints. The primary outcome of our study is the Kessler Psychological Distress Scale 6 (K6), which is a standardised self-reported outcome assessment tool used to measure global non-specific psychological distress with questions relating to depressive and anxiety symptoms. We selected the K6 for its brevity and validation in community-based samples ranging from subclinical to clinical distress [41, 42]. Although floor effects are a possibility in individuals without any significant distress, our preliminary assessments and data from 7 Cups suggest that a noteworthy proportion of participants have mild to moderate distress, making the K6 a pragmatic primary outcome measure. The primary endpoint of the study is the scores of the K6 assessment instrument, which consists of 6 items asking participants to rate their emotional states on a 5-point Likert scale ranging from ‘All the time’ to ‘None of the time’. The K6 is a widely used and accepted assessment instrument for measuring mental health-related intervention outcomes with confirmed reliability and validity. The K6 has good psychometric properties in the general population and is considered reliable with excellent internal consistency reliability of 0.89 [41, 42].

**Table 1:** Measures and timepoints

Measure	Time point			References
	Pre	Post	Follow-Up (Post + 2 weeks)	
K6	x	x	x	Cornelius et al., 2013
PSS-4	x	x	x	Warttig et al., 2013
PHQ-4	x	x	x	Kroenke et al., 2003
UMUX-LITE		x*		Finstrad, 2010
MDMQ (4-item)	x	x	x	Steyer et al., 1997
Qualitative questions		x		
Sociodemo- graphic and other general information	x			

Abbreviations: K6, Kessler Psychological Distress Scale 6; PSS-4, Perceived Stress Scale-4; PHQ, Patient Health Questionnaire; UMUX-LITE, Usability Metric for User Experience; MDMQ, Multidimensional Mood State Questionnaire

*is not completed by subjects in waitlist condition

The secondary outcomes of the study are the Perceived Stress Scale 4 (PSS-4) [43], the 4-item Patient Health Questionnaire (PHQ-4) [44], the 2-item Generalised Anxiety Disorder Questionnaire (GAD-2) [44], and the Multidimensional Mood State Questionnaire (MDMQ, good-bad subscale) (English-language version of MDBF) [45]. Outcome data are collected through self-reported questionnaires and will be analysed to assess the effectiveness and feasibility of the intervention. In addition, a qualitative questionnaire is used to gather further information, feedback, and details about the experience and perceived quality of the intervention [46–48] (see Appendix A for a full list of qualitative and sociodemographic questions). Sociodemographic data are gathered during the baseline assessment for all participants and include age, gender, country of living, and level of education. Participants also complete questions to provide further details of any other interventions used during the intervention.

### Sample size

According to Bilingham et al., (2013) [49] sample size justification is very important, but power calculations may not be appropriate for feasibility studies. Following the recommendations of Gallinat et al., (2018) [50] and Leon, Davis and Kraemer (2011) [51], a sample size of 50 per group, considering potential attrition of 25%, is sufficient for feasibility studies. This sample size allows us to assess feasibility in online family, e-health, and mental health trials for recruitment capabilities, data collection and randomization procedures, participant flow, adherence, and acceptability [49–53] and is still sufficiently large to estimate rough variance for continuous outcomes and trends in expected direction and implementation of outcome measures [49]. We acknowledge that an exploratory study with a small sample size will need to be interpreted cautiously [51]. We thus aim to recruit *N* = 200 participants: 100 *members* (50 in the ST-informed online self-help condition and 50 in the waitlist) and 100 *listeners* (50 in the ST-informed paraprofessional training condition and 50 in the waitlist). Therefore, our targeted sample size of 200 participants should be viewed as supporting an *exploratory* aim. Future trials with a larger sample size will allow a more precise estimation of effect sizes.

### Recruitment

Recruitment for this study spans from 30-08-2023 to 31-03-2024 on the 7 Cups platform (extended from the original end date of 14-02-2024 due to lower-than-anticipated enrolment). During the recruitment period, a systematic process is adopted to enrol patients into the study. The complete informed consent is provided electronically before the beginning of the growth path, listener training or waitlist conditions without any deception. We recruit participants for the OSI/growth path intervention among those members who have signed up for the platform but are not registered or trained for ‘listener’ status. Participants for the paraprofessional/listener training intervention are recruited among those users who have registered as and trained for ‘listener’ status (i.e., volunteering as paraprofessionals on the platform to engage in synchronous text messaging with users seeking emotional support). We use advertisements to attract eligible users and listeners to participate in the study. All members and listeners are screened by the 7 Cups HOR according to the inclusion criteria described above. All members and listeners who are considered eligible are invited to participate in the study by the 7 Cups HOR through an announcement (Appendix B) with a full description of the study. Members and listeners need to provide informed consent to participate in the study (Appendix C) and complete a unique identifying code (UIC), which is a self-generated code that a participant generates by using the first two letters of their mother’s name, the day on which they were born, and the first two letters of the town/city in which they lived at the time of UIC generation (e.g., Mary, 02, Berlin; UIC = ma02be). Participants can withdraw from participation or request that their data be deleted by contacting the 7 Cups HOR.

### Methods, assignment of interventions

#### Allocation

The gold standard of double blinding is not applicable here due to the nature of the intervention, as participants inherently know which condition they are assigned to. Regarding the study team, there are two parts: one within 7 Cups (HOR) and one outside. The HOR team has access to participants, can allocate them, and send messages and links based on their assigned condition. The 7 Cups HOR compiles a list of all members and another list of all listeners who respond to the invitation, provide consent, and complete the UIC. These lists are securely stored on the 7 Cups server and only accessible to the 7 Cups HOR. The 7 Cups HOR provides these two lists of UICs, one for members and one for listeners, to the external team. The external team generates a randomization sequence using unique participant codes and passes this list to the HOR team. Thus, the external team has no immediate knowledge of allocation, and although the HOR team needs to know for procedural aspects, they are blinded to the randomization process. We utilise a random sequence generator to assign member and listener participants to the growth path or waitlist condition and the listener training or waitlist, respectively. The allocation sequence is generated using a computer-based tool provided by Sealed Envelope Ltd. This tool, titled “Create a blocked randomisation list,” was accessed online on August 29, 2023. The sequence was created with a seed of 21 for the list of members and seed of 23 for the list of listeners, and block sizes were set at 50, 100, 150, and 200, resulting in a total list size of 200 for members and 200 for listeners. This method reduces sequence predictability for the 7 Cups HOR who has direct contact with participants. The lists of assigned UICs are sent to the 7 Cups HOR, who messages participants links to either the growth path or waitlist assessments, and listener participants to either the listener training or waitlist assessments, as appropriate. Participants in the member and listener waitlist conditions do not receive any intervention until the waitlist assessments are completed. Upon completion of the assessments, waitlist participants are sent the links to the growth path or listener training for the waitlist members and listeners, respectively. A subsample of participant pairs is selected with each pair consisting of 1 listener and 1 member. The listeners have completed the ST-informed listener training and support their assigned member who is working through the OSI using the chat/messaging system on 7 Cups. For data analysis, the statistician is outside 7 Cups and unaware of allocation issues. The dataset includes allocation information for analysis, but the final data analysis will be performed on a coded dataset once data collection is complete. Regarding assessor bias, our assessments are conducted fully digitally and online without direct human involvement, eliminating the risk of assessor bias. For qualitative analysis, there may be a risk of bias if raters know about allocation. Therefore, we will have independent raters who are unaware of participants’ allocation. This approach is adopted to ensure the integrity and transparency of the study while acknowledging the limitations posed by the online setting.

### Data collection, management and analysis

The study employs a pre-post-follow-up design for the assessments. These assessments are facilitated through LimeSurvey, a General Data Protection Regulations (GDPR) compliant tool. The links for the t0 pre-assessment are sent to participants after being assigned to their respective conditions by the 7 Cups HOR. Our research team and the 7 Cups HOR monitor the progress of the participants and send them a link to complete the t1 post-assessment survey upon completion of the growth path, listener training, or 4 weeks into the waitlist condition. Participants who stop progressing on the growth path, listener training, or assessments for more than a week are sent reminders by the 7 Cups HOR. A link for the t2 follow-up assessment is sent two weeks after the completion of the t1 assessment. After completing the t2 assessment, the member and listener waitlist participants are sent the link to the growth path or listener training, respectively. If participants want to opt out of the study or request their assessment data to be deleted, they are instructed to contact the 7 Cups HOR or the Data Protection Officer at the IPU. After data cleaning, all participant UICs will be deleted, resulting in a fully anonymized dataset for further analysis.

### Data management

The study is conducted through the online platform 7 Cups, which has all the necessary infrastructure to provide the growth path, listener training, and participant communication. After questionnaire and assessment data are collected via LimeSurvey, it is transferred to a secure database on the IPU servers, accessible only to authorised personnel. Routine backups are conducted to ensure data safety and confidentiality. Data handling by 7 Cups, IPU, and LimeSurvey complies with the GDPR and is supervised by the 7 Cups HOR and the data protection officer of IPU. Throughout the study, general data management, including exclusions, recruitment, dropout, communication, and participant rate is meticulously recorded. Data collection commenced on 30-08-2023 and last patient/last visit is projected to conclude on 31-03-2024. Double data entry will not be conducted as all data for this study is collected digitally and digital data entries are restricted to valid entries only.

### Statistical methods

For statistical analyses and handling of missing data, descriptive statistics and estimation of intervention effects are planned following recognised guidelines. Appropriate regression methods shall be used, depending on the outcome parameters’ distributional characteristics. To estimate intervention effects, we intend to conduct generalised linear mixed models of primary, and secondary parameters, adjusted for potential covariates (e.g., gender, age categories, socioeconomic status) as fixed effects. The exact choice of regression method shall consider the distributional characteristics of the outcome parameters of interest. Concerning the management of missing data, we aim to minimise bias through thorough planning and active data review. A differentiation shall be made between missing data due to partial participation and loss to follow-up, and appropriate statistical methods shall be considered to address missingness.

### Monitoring

The Principal Investigator (PI) assumes responsibility for several key aspects of the study. The PI is in charge of any interim analyses and stopping guidelines, maintaining exclusive access to these interim results and making the final decision to terminate the trial. The PI also establishes plans for collecting, assessing, reporting, and managing solicited and spontaneously reported adverse events and other unintended effects of the intervention or participation. This approach upholds the integrity and transparency of the study while acknowledging the responsibilities of the PI.

This study does not include a formal, dedicated measure of adverse events. However, in line with 7 Cups safety protocols, any participant presenting signs of self-harm risk or crisis is escalated to crisis resources and local emergency services if needed. The PI monitors all communications from the 7 Cups Head of Research (HOR) related to adverse events. If serious adverse events occur, they are documented and reviewed as part of the ethical oversight.

### Ethics

In terms of ethics and dissemination, the study is designed with a strong emphasis on ethical considerations and transparency. Although no formal Data Monitoring Committee was established due to the low-risk nature of the intervention, provisions are in place for the premature termination of the study under specific circumstances, such as insufficient participant recruitment, significant changes in 7 Cups practices, or early evidence of harm or benefit from the growth path or listener training. Training was completed by all authorised personnel who are involved in the study to ensure responsibilities, and protocols are clear and understood. Despite the minimal anticipated risk, the study thoroughly assesses any potential harm. Serious Adverse Events (SAEs) that occur during the study, including those related to suicide attempts or completed suicide, are continuously monitored and responded to by 7 Cups. Internal audits are carried out to verify all procedures, including recruitment, consent, enrolment, and data collection.

Data confidentiality and secure coding are prioritised. Participants’ data are only accessible to authorised personnel and securely stored. The complete dataset, once finalised, will be transferred to the principal investigator, with limited access granted to other IPU members for analysis.

### Dissemination plans

We intend to publish the key results of the study followed by publications focusing on selected aspects of the study in international peer-reviewed journals. Furthermore, we intend to communicate key results to the public via the 7 Cups platform following data analyses. Public access to the full protocol is provided by this manuscript. Public access to participant-level datasets is not intended. Access to statistical codes is intended to be provided on request. This protocol was written following the SPIRIT protocol guidance.

## Discussion

Despite the great need for mental health interventions, there is limited research available showing the efficacy of digitally delivered ST-informed interventions [54]. This study represents an exploratory RCT that primarily aims to assess feasibility and gather preliminary efficacy data regarding digitally delivered ST-informed interventions. This study additionally aims to explore the challenges, and user experience of novel, digital interventions on a widely accessible platform (7 Cups). By investigating a digital self-help and paraprofessional program informed by systemic therapy principles, this study aims to take a step towards enhancing global mental health support. Our aim in investigating paraprofessionals’ outcomes is to assess whether learning ST-informed techniques may benefit listeners’ mental health. A broader question—whether this guidance also improves member outcomes beyond unguided OSI—would require a separate design. Future research with larger samples is encouraged to address how paraprofessional guidance modifies member outcomes relative to purely self-guided interventions. We hypothesize that intervention group members will show greater reductions in mental health distress compared to control groups. The strengths of this research include its theory-based intervention and consideration of multiple covariates. A potential limitation is that participants with very low baseline distress may not demonstrate measurable change on the K6 or other symptom-based measures. Future research might include dedicated measures of positive wellbeing or functioning to better capture change in subclinical populations. The findings may contribute to discussions on digital mental health strategies to alleviate psychological distress on a large scale that overcomes common barriers to mental health interventions such as cost. This project is an exploratory approach to evaluate feasibility and preliminary efficacy. Claims about long-term effectiveness are beyond its scope, and larger-scale trials would be necessary to confirm or refute efficacy on a broader scale. While this study is exploratory and not fully powered, it may serve as a starting point to shape larger, more definitive evaluations. It could also contribute to the ongoing dialogue about how digital mental health interventions are designed and scaled. In future work, employing a formal implementation science framework would further clarify how ST-informed digital interventions can be effectively deployed at scale.

## Electronic supplementary material

Below is the link to the electronic supplementary material.

Supplementary Material 1

Supplementary Material 2

## Data Availability

No datasets were generated or analysed during the current study.

## Abbreviations

G-BA: Gemeinsamer Bundesausschuss
GAD-2: Generalised Anxiety Disorder Questionnaire 2-Item
GDPR: General Data Protection Regulation
HOR: Head of Research
IQWiG: Institut für Qualität und Wirtschaftlichkeit im Gesundheitswesen
IPU: International Psychoanalytic University
K6: Kessler Psychological Distress Scale 6-Item
K10: Kessler Psychological Distress Scale 10-Item
MDBF: Mehrdimensionaler Befindlichkeitsfragebogen
MDMQ: Multidimensional Mood State Questionnaire
OSI: Online Self-help Intervention
PI: Principal Investigator
PHQ-4: Patient Health Questionnaire 4-Item
PSS-4: Perceived Stress Scale 4-Item
RCT: Randomised Controlled Trial
ST: Systemic Psychotherapy
UIC: Unique Identifying Code
UMUX-LITE: Two-item questionnaire based on the Usability Metric for User Experience (UMUX)

## Declarations

### Generative AI and AI-assisted technologies in the writing process

During the preparation of this work, the author(s) used Co-pilot in order to help prepare the outline for the protocol. After using this tool/service, the author(s) reviewed and edited the content as needed and take(s) full responsibility for the content of the publication.

## References

[1] Baumel A, Tinkelman A, Mathur N, Kane JM. Digital Peer-Support platform (7Cups) as an adjunct treatment for women with postpartum depression: feasibility, acceptability, and preliminary efficacy study. JMIR MHealth UHealth. 2018;6(2):e38. 29439944 10.2196/mhealth.9482 PMC5829455

[2] Carr A. Couple therapy, family therapy and systemic interventions for adult-focused problems: the current evidence base. J Fam Ther. 2019;41(4):492–536.

[3] Helps S, Le Coyte Grinney M. Synchronous digital couple and family psychotherapy: a meta-narrative review. J Fam Ther. 2021;43(2):185–214.

[4] De Boer K, Muir SD, Silva SSM, Nedeljkovic M, Seabrook E, Thomas N, et al. Videoconferencing psychotherapy for couples and families: A systematic review. J Marital Fam Ther. 2021;47(2):259–88. 33837968 10.1111/jmft.12518

[5] McLean SA, Booth AT, Schnabel A, Wright BJ, Painter FL, McIntosh JE. Exploring the efficacy of telehealth for family therapy through systematic, Meta-analytic, and qualitative evidence. Clin Child Fam Psychol Rev. 2021;24(2):244–66. 33492545 10.1007/s10567-020-00340-2 PMC7829321

[6] IQWiG. Systemische Therapie bei Erwachsenen als Psychotherapieverfahren, Abschlussbericht, Auftrag N14-02. [Internet]. Institut für Qualität und Wirtschaftlichkeit im Gesundheitswesen; 2017 May [cited 2021 Sep 1]. Report No.: 513. Available from: https://www.iqwig.de/download/n14-02_abschlussbericht_systemische-therapie-bei-erwachsenen_v1-0.pdf

[7] Riedinger V, Pinquart M, Teubert D. Effects of systemic therapy on mental health of children and adolescents: A Meta-Analysis. J Clin Child Adolesc Psychol. 2017;46(6):880–94. 26467300 10.1080/15374416.2015.1063427

[8] Stratton P, Lask J. The development of systemic family therapy for changing times in the united Kingdom. Contemp Fam Ther. 2013;35(2):257–74.

[9] Von Sydow K, Retzlaff R, Beher S, Haun MW, Schweitzer J. The efficacy of systemic therapy for childhood and adolescent externalizing disorders: A systematic review of 47 RCT. Fam Process. 2013;52(4):576–618. 24102196 10.1111/famp.12047

[10] Retzlaff R. Systemische Therapie– Fallkonzeption, Therapieplanung, Antragsverfahren. Carl-Auer verlag. Germany: Heidelberg; 2023.

[11] Levold T, Wirsching M. Systemische therapie und Beratung– das Große lehrbuch. Carl-Auer verlag, Germany: Heidelberg; 2023.

[12] Lorås L, Bertrando P, Ness O. Researching systemic therapy history: in search of a definition. J Fam Psychother. 2017;28(2):134–49.

[13] Schiepek G. Die grundlagen der Systemischen therapie: theorie, praxis, forschung. Göttingen: Vandenhoeck & Ruprecht; 1999. p. 450.

[14] Retzlaff R, Von Sydow K, Beher S, Haun MW, Schweitzer J. The efficacy of systemic therapy for internalizing and other disorders of childhood and adolescence: A systematic review of 38 randomized trials. Fam Process. 2013;52(4):619–52. 24329407 10.1111/famp.12041

[15] European Association for Psychotherapy. https://www.europsyche.org. Accessed 20 February 2025.

[16] Baumel A, Pawar A, Kane JM, Correll CU. Digital parent training for children with disruptive behaviors: systematic review and Meta-Analysis of randomized trials. J Child Adolesc Psychopharmacol. 2016;26(8):740–9. 27286325 10.1089/cap.2016.0048

[17] Sourander A, McGrath PJ, Ristkari T, Cunningham C, Huttunen J, Lingley-Pottie P, et al. Internet-Assisted parent training intervention for disruptive behavior in 4-Year-Old children: A randomized clinical trial. JAMA Psychiatry. 2016;73(4):378. 26913614 10.1001/jamapsychiatry.2015.3411

[18] Pal K, Dack C, Ross J, Michie S, May C, Stevenson F, et al. Digital health interventions for adults with type 2 diabetes: qualitative study of patient perspectives on diabetes Self-Management education and support. J Med Internet Res. 2018;20(2):e40. 29463488 10.2196/jmir.8439 PMC5931778

[19] Syed ST, Gerber BS, Sharp LK. Traveling towards disease: transportation barriers to health care access. J Community Health. 2013;38(5):976–93. 23543372 10.1007/s10900-013-9681-1 PMC4265215

[20] Orsolini L, Pompili S, Salvi V, Volpe U. A systematic review on telemental health in youth mental health: focus on anxiety, depression and Obsessive-Compulsive disorder. Med (Mex). 2021;57(8):793. 10.3390/medicina57080793 PMC8398756 34440999

[21] Glynn SM, Randolph ET, Garrick T, Lui A. A proof of concept trial of an online psychoeducational program for relatives of both veterans and civilians living with schizophrenia. Psychiatr Rehabil J. 2010;278–87. 10.2975/33.4.2010.278.287 20374986

[22] Markman HJ, Hawkins AJ, Stanley SM, Halford WK, Rhoades G. Helping couples achieve relationship success: A decade of progress in couple relationship education research and practice, 2010– 2019. J Marital Fam Ther. 2021;00:1–32. 10.1111/jmft.12565 PMC9298911 34783038

[23] Narad ME, Minich N, Taylor HG, Kirkwood MW, Brown TM, Stancin T, Wade SL. Effects of a web-based intervention on family functioning following pediatric traumatic brain injury. J Dev Behav Pediatr. 2015;36:700–7. 26461100 10.1097/DBP.0000000000000208 PMC4635046

[24] Cuijpers P, Noma H, Karyotaki E, Cipriani A, Furukawa TA. Effectiveness and acceptability of cognitive behavior therapy delivery formats in adults with depression: A network Meta-analysis. JAMA Psychiatry. 2019;76(7):700. 30994877 10.1001/jamapsychiatry.2019.0268 PMC6583673

[25] Pauley D, Cuijpers P, Papola D, Miguel C, Karyotaki E. Two decades of digital interventions for anxiety disorders: a systematic review and meta-analysis of treatment effectiveness. Psychol Med. 2021;1–13. 10.1017/S0033291721001999 PMC9899576 34047264

[26] Karyotaki E, Riper H, Twisk J, Hoogendoorn A, Kleiboer A, Mira A, et al. Efficacy of Self-guided Internet-Based cognitive behavioral therapy in the treatment of depressive symptoms: A Meta-analysis of individual participant data. JAMA Psychiatry. 2017;74(4):351. 28241179 10.1001/jamapsychiatry.2017.0044

[27] Torok M, Han J, Baker S, Werner-Seidler A, Wong I, Larsen ME, et al. Suicide prevention using self-guided digital interventions: a systematic review and meta-analysis of randomised controlled trials. Lancet Digit Health. 2020;2(1):e25–36. 33328037 10.1016/S2589-7500(19)30199-2

[28] Andersson G. Internet interventions: past, present and future. Internet Interv. 2018;12:181–8. 30135782 10.1016/j.invent.2018.03.008 PMC6096319

[29] Karyotaki E, Van Ballegooijen W. Digital self-help interventions for suicidal ideation and behaviour. Lancet Digit Health. 2020;2(1):e4–5. 33328038 10.1016/S2589-7500(19)30215-8

[30] Fairburn CG, Patel V. The impact of digital technology on psychological treatments and their dissemination. FOCUS. 2018;16(4):449–55. 32021582 10.1176/appi.focus.16405 PMC6996082

[31] Erbe D, Eichert HC, Riper H, Ebert DD. Blending Face-to-Face and Internet-Based interventions for the treatment of mental disorders in adults: systematic review. J Med Internet Res. 2017;19(9):e306. 28916506 10.2196/jmir.6588 PMC5622288

[32] Ebert DD, Erbe D. Internetbasierte psychologische Interventionen. In: Berking M, Rief W, editors. Klinische Psychologie und Psychotherapie für Bachelor [Internet]. Berlin, Heidelberg: Springer Berlin Heidelberg; 2012 [cited 2024 Feb 2]. pp. 131–40. (Springer-Lehrbuch; vol. 5024). Available from: http://link.springer.com/10.1007/978-3-642-25523-6_12

[33] Sin J, Galeazzi G, McGregor E, Collom J, Taylor A, Barrett B, et al. Digital interventions for screening and treating common mental disorders or symptoms of common mental illness in adults: systematic review and Meta-analysis. J Med Internet Res. 2020;22(9):e20581. 32876577 10.2196/20581 PMC7495259

[34] Ng MM, Firth J, Minen M, Torous J. User engagement in mental health apps: A review of measurement, reporting, and validity. Psychiatr Serv. 2019;70(7):538–44. 30914003 10.1176/appi.ps.201800519 PMC6839109

[35] Torous J, Lipschitz J, Ng M, Firth J. Dropout rates in clinical trials of smartphone apps for depressive symptoms: A systematic review and meta-analysis. J Affect Disord. 2020;263:413–9. 31969272 10.1016/j.jad.2019.11.167

[36] Leung C, Pei J, Hudec K, Shams F, Munthali R, Vigo D. The effects of nonclinician guidance on effectiveness and process outcomes in digital mental health interventions: systematic review and Meta-analysis. J Med Internet Res. 2022;24(6):e36004. 35511463 10.2196/36004 PMC9244656

[37] Rosenberg NE, Artman-Meeker K, Kelly E, Yang X. The effects of a Bug-in-Ear coaching package on implementation of incidental teaching by paraprofessionals in a K-12 school. J Behav Educ. 2020;29(2):409–32.

[38] Barnett ML, Gonzalez A, Miranda J, Chavira DA, Lau AS. Mobilizing community health workers to address mental health disparities for underserved populations: A systematic review. Adm Policy Ment Health Ment Health Serv Res. 2018;45(2):195–211. 10.1007/s10488-017-0815-0 PMC5803443 28730278

[39] Singla DR, Kohrt BA, Murray LK, Anand A, Chorpita BF, Patel V. Psychological treatments for the world: lessons from Low- and Middle-Income countries. Annu Rev Clin Psychol. 2017;13(1):149–81. 28482687 10.1146/annurev-clinpsy-032816-045217 PMC5506549

[40] Naslund JA, Shidhaye R, Patel V. Digital technology for Building capacity of nonspecialist health workers for task sharing and scaling up mental health care globally. Harv Rev Psychiatry. 2019;27(3):181–92. 30958400 10.1097/HRP.0000000000000217 PMC6517068

[41] Cornelius BL, Groothoff JW, Van Der Klink JJ, Brouwer S. The performance of the K10, K6 and GHQ-12 to screen for present state DSM-IV disorders among disability claimants. BMC Public Health. 2013;13(1):128. 23402478 10.1186/1471-2458-13-128 PMC3575398

[42] Kessler RC, Andrews G, Colpe LJ, Hiripi E, Mroczek DK, Normand SLT, et al. Short screening scales to monitor population prevalences and trends in non-specific psychological distress. Psychol Med. 2002;32(6):959–76. 12214795 10.1017/s0033291702006074

[43] Warttig SL, Forshaw MJ, South J, White AK. New, normative, English-sample data for the short form perceived stress scale (PSS-4). J Health Psychol. 2013;18(12):1617–28. 24155195 10.1177/1359105313508346

[44] Kroenke K, Spitzer RL, Williams JBW, Lowe B. An Ultra-Brief screening scale for anxiety and depression: the PHQ-4. Psychosomatics. 2009;50(6):613–21. 19996233 10.1176/appi.psy.50.6.613

[45] Steyer R, Schwenkmezger P, Notz P, Eid M. Der mehrdimensionale befindlichkeitsfragebogen MDBF [multidimensional mood questionnaire]. Göttingen, Germany: Hogrefe; 1997.

[46] Revicki DA. Patient assessment of treatment satisfaction: methods and practical issues. Gut. 2004;53(suppl4):iv40–4. 15082613 10.1136/gut.2003.034322 PMC1867784

[47] Ghani MA, Brown SE, Khan F, Wickersham JA, Lim SH, Dhaliwal SK, et al. An exploratory qualitative assessment of self-reported treatment outcomes and satisfaction among patients accessing an innovative voluntary drug treatment centre in Malaysia. Int J Drug Policy. 2015;26(2):175–82. 25577322 10.1016/j.drugpo.2014.10.002 PMC4291539

[48] Johnsen AT, Petersen MA, Pedersen L, Groenvold M. Development and initial validation of the Three-Levels-of-Needs questionnaire for self-assessment of palliative needs in patients with cancer. J Pain Symptom Manage. 2011;41(6):1025–39. 21306865 10.1016/j.jpainsymman.2010.08.013

[49] Billingham SA, Whitehead AL, Julious SA. An audit of sample sizes for pilot and feasibility trials being undertaken in the united Kingdom registered in the united Kingdom clinical research network database. BMC Med Res Methodol. 2013;13:104. 23961782 10.1186/1471-2288-13-104 PMC3765378

[50] Gallinat C, Moessner M, Haenssle HA, Winkler JK, Backenstrass M, Bauer S, SaveMySkin. An Internet-based self-help intervention for skin picking. Study protocol for a randomized pilot study. Contemp Clin Trials Commun. 2018;13:100315. 31011654 10.1016/j.conctc.2018.100315 PMC6465578

[51] Leon AC, Davis LL, Kraemer HC. The role and interpretation of pilot studies in clinical research. J Psychiatr Res. 2011;45(5):626–9. 21035130 10.1016/j.jpsychires.2010.10.008 PMC3081994

[52] Whitehead AL, Julious SA, Cooper CL, Campbell MJ. Estimating the sample size for a pilot randomised trial to minimise the overall trial sample size for the external pilot and main trial for a continuous outcome variable. Stat Methods Med Res. 2016;25(3):1057–73. 26092476 10.1177/0962280215588241 PMC4876429

[53] Teresi JA, Yu X, Stewart AL, Hays RD. Guidelines for designing and evaluating feasibility pilot studies. Med Care. 2022;60(1):95–103. 34812790 10.1097/MLR.0000000000001664 PMC8849521

[54] Erasmus P, Borrmann M, Becker J, Kuchinke L, Meinlschmidt G. Comparing digital versus face-to-face delivery ofsystemic psychotherapy interventions: systematic review and meta-analysis of randomized controlled trials. Interact J Med Res. 2025;14:e46441. 10.2196/46441 PMC11894358 39993307

